# Liposarcome myxoïde primitif du médiastin moyen

**DOI:** 10.11604/pamj.2014.19.66.5276

**Published:** 2014-09-24

**Authors:** Rachid Marouf, Ihssan Alloubi

**Affiliations:** 1 Service de Chirurgie Thoracique, CHU Mohammed VI, Oujda, Maroc

**Keywords:** Liposarcome primitif, myxoïde, médiastin, chirurgie, pronostic, primitive liposarcoma, myxoid, mediastinum, surgery, prognosis

## Abstract

Les liposarcomes “LPS” myxoïdes sont des tumeurs rares, notamment dans leur localisation médiastinale. Nous rapportons un cas d'un liposarcome myxoïde du médiastin moyen, chez un patient de 69 ans, sans passé pathologique particulier et sans signes d'appels respiratoires. Une radiographie thoracique faite lors d'un bilan préopératoire montre un élargissement médiastinal dont le scanner thoracique le rattache à une tumeur hétéro dense, siège de quelques zones de densité graisseuse au niveau du médiastin moyen. Malgré son volume, cette masse parait de contours nets, sans envahissement ni compression des structures adjacentes. La ponction biopsie transpariétale scanno-guidée est compatible avec un liposarcome myxoïde; Une exérèse chirurgicale complète est réalisée. Après 24 mois de surveillance, le patient ne présente pas de signe de récidive. Le LPS myxoïde du médiastin est une entité rarissime et quelques cas sporadiques ont été reportés dans la littérature. La chirurgie parait être le traitement de choix. La radiothérapie et la chimiothérapie gardent leur place dans des indications bien particulières.

## Introduction

Les liposarcomes (LPS) sont des tumeurs relativement rares, représentant environ 15 à 20% de tous les sarcomes. Ils surviennent le plus souvent dans les membres inférieurs (75%) et moins fréquemment dans le rétropéritoine. La localisation médiastinale reste exceptionnelle, comprenant moins de 1% de toutes les tumeurs du médiastin [1]. Nous rapportons le cas d´un liposarcome myxoïde du médiastin moyen avec revue de la littérature.

## Patient et observation

**Figure 1:**
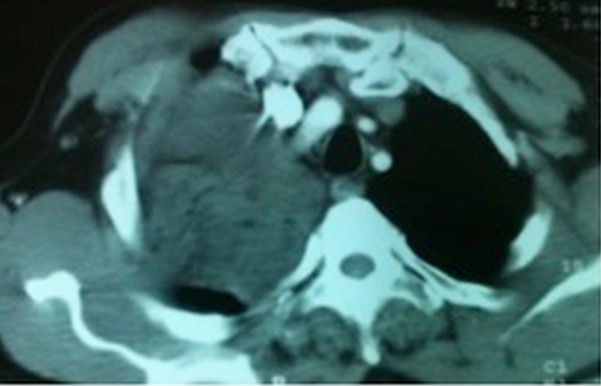
TDM thoracique: masse tissulaire hétéro-dense occupant l'hémi thorax droit, de 10x9x6cm, siège de zones de densité graisseuse, et refoulant la VCS, et arrivant au contact de la trachée, l’œsophage, et aux corps vertébraux sans lyse osseuse

**Figure 2:**
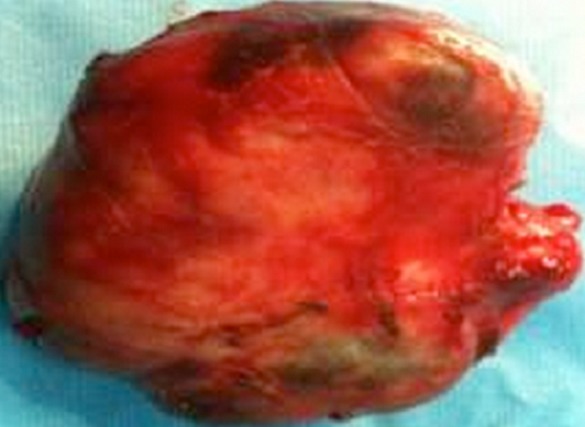
Pièce d'exérèse chirurgicale

Un patient âgé de 69 ans, sans passé pathologique particulier, non connu tabagique, et chez qui on découvre fortuitement, sur une radiographie thoracique faite lors d´un bilan préopératoire, une opacité hydrique bien circonscrite, à point de départ médiastinal avec une composante endothoracique droite. L'examen clinique est sans particularité. Le scanner thoracique montre la présence, au niveau du médiastin moyen d'une masse tissulaire hétéro dense, assez bien limitée, siège de quelques zones de densité graisseuse, de 10x9x6cm, cette masse refoule la VCS, et arrive au contact de la paroi latérale droite de la trachée, de l’œsophage, de l'artère pulmonaire droite et des hémicorps vertébraux de D1-D2 sans lyse osseuse décelable (Figure 1). L´endoscopie bronchique ne montre qu´une simple compression extrinsèque de la bronche principale droite. Le bilan d´extension (TDM cérébrale et abdominale) est négatif. La ponction biopsie Trans-pariétale scanno-guide'e révèle un aspect de liposarcome myxoïde. Une exploration chirurgicale est effectuée. La voie d'abord est une stérnotomie partielle associée à une thoracotomie antérolatérale droite (hémi-clamshell droit). Une exérèse chirurgicale en monobloc d'une énorme masse médiastinale de consistance tissulaire polylobée, bien encapsulée, adhérente à la plèvre pariétale au niveau apical droit obligeant le passage en extra pleural, sans envahissement des éléments du médiastin (Figure 2). Les suites postopératoires sont simples. L’étude anatomo-pathologique met en évidence une masse polylobée bien limitée, encapsulée, pesant 370g, mesurant 10,5x8x5, 5cm, de type myxoïde bien différenciée, d'aspect jaunâtre avec par place, présence d'un aspect blanchâtre fibreux. Aspect morphologique d'un liposarcome myxoïde d'exérèse complète. Remaniement fibro-congestifs de la plèvre. Après 24 mois de surveillance, on ne note pas de récidive locorégionale ni de métastase.

## Discussion

Les liposarcomes sont des sarcomes des tissus mous avec différenciation adipocytaire. Ils représentent environ 15% des tumeurs malignes des tissus mous de l'adulte et la variété myxoïde représente 40 à 50% des LPS, toutes localisations confondues [1, 2]. Ils surviennent généralement dans les membres inférieurs (75%) et moins fréquemment dans le rétro péritoine [2]. Les liposarcomes primaires du médiastin, avec moins de 200 cas rapportés dans la littérature à ce jour, sont des tumeurs extrêmement rares, représentant moins de 1% de toutes les tumeurs du médiastin [2]. Les LPS myxoïdes atteignent généralement l'adulte avec un pic d'incidence au cours de la 4ème et la 5^ème^ décennie. L’étude réalisée par « Hahn» montre que l’âge moyen de ses patients lors du diagnostic été 58 ans [3]. Le LPS myxoïde peut être asymptomatique et de découverte radiologique dans environ 12% des cas [4]. Il devient symptomatique par la compression qu'il entraine sur les structures médiastinales alors qu'il a acquis un volume important, notamment sur l'axe trachéo-bronchique, le cœur et les vaisseaux, et l’œsophage. Ce qui a comme conséquence une limitation fonctionnelle tel que la toux, la dyspnée, le sifflement, le wheezing, la dysphagie, l'angine de poitrine ou le syndrome cave supérieur [4].

Les images radiologiques du LPS ne sont pas spécifiques mais suggestives du diagnostic. La radiographie thoracique montre en général un élargissement médiastinal, ou une déviation de l'axe trachéal ou des vaisseaux [2]. Au scanner thoracique: le LPS médiastinal prend l´aspect d'une masse inhomogène, mal limitée, contenant des zones de densité graisseuse et d´autres de densité solide. De faibles valeurs d´atténuation entre -50 et -150 Unité Hounsfield (HU) sont compatibles avec un tissu composé de matière grasse, des valeurs plus élevées sont liées à la présence de la nécrose, ou des tissus mous. L´imagerie par résonance magnétique (IRM) peut apporter des éléments déterminants dans la résécabilité, et montrer avec précision les limites de la tumeur avec les éléments du médiastin, son extension locorégionale. Les diagnostiques différentiels sont les lipomes, les thymo-lipomes, les tératomes, les lymphomes, et les tumeurs des cellules germinales [2, 5]. Le diagnostic du LPS myxoïde est histologique, par la découverte d'une tumeur à stroma myxoïde, présentant une vascularisation avec un aspect caractéristique « branché » constitué de vaisseaux grêles et la présence le lipoblastes, l'absence d'atypie nucléaire, et de rares mitoses [1].

La classification de l´OMS reconnait 5 catégories de LPS qui se repartissent de la façon suivante [2]: LPS bien différencié; LPS de type adipocytaire (lipome atypique); LPS sclérosant; LPS inflammatoire LPS à cellules fusiformes; LPS dédifférencié; LPS myxoïdes à cellules rondes; LPS pléomorphes LPS mixtes combinés. Les LPS myxoïdes et bien différenciés sont des sarcomes de faible grade de malignité, le plus souvent encapsulées et métastasient rarement. Alors que les LPS à cellules rondes, pléomorphes et dédifférenciés sont de haute malignité avec une tendance à la récidive locale et métastasient précocement [1, 4]. En raison de la faible incidence des LPS à localisation médiastinale, les stratégies thérapeutiques sont extrapolées à partir des tumeurs similaires dans d´autres endroits.

Le traitement optimal des LPS myxoïdes est la résection chirurgicale radicale avec des marges négatives (R0), Un centimètre est choisi comme seuil dans certaines études. Par ailleurs, ces tumeurs ont tendance à envahir les structures adjacentes rendant leur exérèse complète difficile [3, 6]. La chirurgie reste également le traitement de choix des récidives locales [6]. La chirurgie de réduction ou “Debulking” garde son indication à titre palliatif pour les tumeurs invasives et non résécables en totalité, elle entraîne souvent un soulagement des symptômes [2]. L'intérêt d'une radiothérapie complémentaire systématique en cas de résection complète est controversé. Elle reste réservée aux formes peu différenciées, aux tumeurs non résécables et aux tumeurs récidivantes [1, 7]. La chimiothérapie peut être utilisée dans les formes métastatiques de LPS myxoïde. En traitement néo adjuvant, elle peut également faciliter une exérèse chirurgicale radicale [1, 8]. La doxorubicine et l´ifosfamide sont les médicaments les plus utilisés en 1ère ligne en chimiothérapie des LPS myxoïdes [9]. La Trabectedin est une option de seconde line [10].

L'intérêt de traitements adjuvants et néo adjuvants dans les LPS myxoïdes médiastinaux est difficile à préciser et mérite des investigations complémentaires pour prouver leur efficacité du fait du petit nombre de séries, et la nature rétrospective des études. Hirai et al. ont rapporté que la radiothérapie et la chimiothérapie sont des modalités thérapeutiques inefficaces en terme de survie [6]. Le type histologique et la résection chirurgicale radicale sont les facteurs qui influencent le comportement et le pronostic des LPS myxoïdes [7]. Enterline et al. ont rapporté que les forme purement myxoïde, et bien différenciées ont le meilleur pronostic avec un taux de survie à 5 ans d´environ 60% par rapport à 10% pour la variété pléomorphe [11]. Dans la série de 103 patients d´Enziger Winslow, les formes bien différenciées ont une survie moyenne à 5 ans de 85% et les formes myxoïdes de 77% [1]. A long terme, un suivi attentif doit être encore fortement recommandé.

## Conclusion

Les liposarcomes myxoïdes du médiastin sont des tumeurs très rares. Le diagnostic de présomption peut être obtenu par des investigations radiologiques et la confirmation est histologique. La chirurgie radicale reste le traitement de choix chez les patients porteurs de liposarcome myxoïde et permet d'obtenir les meilleures survies
